# SARS Risk Perception and Preventive Measures, Singapore and Japan

**DOI:** 10.3201/eid1104.040765

**Published:** 2005-04

**Authors:** David Koh, Ken Takahashi, Meng-Kin Lim, Teppei Imai, Sin-Eng Chia, Feng Qian, Vivian Ng, Calvin Fones

**Affiliations:** * National University of Singapore, Singapore;; † University of Occupational and Environmental Health, Kitakyushu, Japan

**Keywords:** letter, SARS, healthcare workers, social and work impact, risk perception

**To the Editor:** Healthcare workers accounted for 21% of all cases of severe acute respiratory syndrome (SARS) during the 2002–2003 outbreak (*1*). We studied perceptions of risk for SARS infection and preventive measures among healthcare workers in Singapore, who handled cases of SARS and where >41% of the cases occurred among healthcare workers, and in Japan, a SARS-free country.

A self-administered questionnaire was distributed to healthcare workers in various healthcare settings in Singapore (n = 15,025) and Japan (n = 9,978) from May to September 2003. Healthcare workers in Singapore were from 9 primary healthcare hospitals and 9 major institutional healthcare settings, including 3 tertiary hospitals where cases of SARS occurred among healthcare workers, 1 specialized women and children's hospital, 2 community hospitals, and 2 tertiary dental centers. In Japan, study participants were healthcare workers at 7 tertiary-level hospitals distributed throughout Japan. Four of these are university-attached, 2 are municipal hospitals, and 1 is a private hospital.

A total of 10,511 (70% response) and 7,282 (73% response) valid questionnaires were returned in Singapore and Japan, respectively. A total of 43% and 45% of the healthcare workers in Singapore and Japan were nurses; others were doctors, physiotherapists, pharmacists, attendants, cleaning staff, and administrative or clerical staff. In terms of sociodemographic characteristics, the mean ages of the healthcare workers were 36.6 years in Singapore and 35.6 years in Japan, while the gender distribution was 82% female in Singapore and 70% female in Japan, respectively. Approximately half (57% and 45%, respectively) of healthcare workers in Singapore and Japan were married.

A similar proportion (about two thirds) of healthcare workers in both countries felt at great risk of exposure to SARS. However, a higher proportion (76%) was afraid of contracting SARS in Singapore as compared to Japan (55%). Nearly all healthcare workers (96%) in Singapore felt that implementation of protective measures at work was generally effective, and 95% were satisfied with the explanation of their necessity and importance. Slightly fewer (93%) agreed that clear policies and protocols for everyone to follow were in place. In contrast, among Japanese healthcare workers, only 65% agreed that clear policies and protocols were in place, and many fewer (31%) felt that protective measures at work were generally effective (Table).

**Table:** Singaporean and Japanese healthcare workers' risk perceptions about severe acute respiratory syndrome (SARS)

Perceptions	Workers	
	Singapore (n = 10,511),%	Japan (n = 7,282),%
Felt at great risk of exposure to SARS	66	64
Were afraid of contracting SARS	76	55
Felt that protective measures were effective	96	31
Thought that protective measures were necessary and important	95	88
Felt that policies and protocols were clear	93	65
Thought that policies and protocols were implemented	90	50
Felt that recommended measures were adhered to	92	43

As to the national experiences with the SARS outbreak, healthcare workers in Singapore managed 238 cases of SARS, while those in Japan did not encounter any cases. Furthermore, preventive measures were strictly enforced and effective in Singapore, and the outbreak was contained successfully. In contrast, preventive measures were in place in Japan, but workers lacked confidence in an untested system (*2*). These differences are probable explanations for the varying responses in the Singaporean and Japanese healthcare workers. The perceived need for adherence to prescribed measures and willingness to follow protocols were quite different, given the difference in perceived risks. In SARS-free Japan, most healthcare workers were aware that institutional policies and protocols existed, but less than a third were confident of their effectiveness. The degree of implementation and adherence endorsed by healthcare workers was also lower in Japan.

Infections of healthcare workers at the onset of an outbreak may be due to perceptions that recommended policies and measures are unnecessary or excessive. Thus, efforts to educate and communicate the rationale and importance of protective measures may be especially important when outbreaks seem distant and perceived danger is low.

Healthcare workers in both Singapore and Japan perceived a risk for exposure to SARS at work, which reflected the global reach of the illness. However, in Singapore, where cases existed and where the disease was eventually contained, the perceived danger of contracting the illness was higher, and most healthcare workers were reassured by the preventive measures taken, which they viewed as effective. This situation was in contrast to the healthcare workers' perceptions of infection risk and confidence in preventive measures against SARS in Japan, where the measures for infection control were untested.

## References

[1] World Health Organization. WHO consensus document. Geneva: The Organization; 2003.

[2] Kaminota M. Prepare for the return of SARS in this winter [article in Japanese]. Koshu Eisei. 2003;67:826–30.

